# Nanoplatforms: The future of oral cancer treatment

**DOI:** 10.1002/hsr2.1471

**Published:** 2023-08-02

**Authors:** Kalpani Senevirathna, Shalindu M. Jayawickrama, Yovanthi A. Jayasinghe, Karunakalage I. P. Prabani, Kushani Akshala, Ratupaskatiye G. G. R. Pradeep, Hewaratne D. W. T. Damayanthi, Kalani Hettiarachchi, Thinley Dorji, Don E. Lucero‐Prisno, Rajapakse M. G. Rajapakse, Kehinde K. Kanmodi, Ruwan D. Jayasinghe

**Affiliations:** ^1^ Centre for Research in Oral Cancer, Faculty of Dental Sciences University of Peradeniya Peradeniya Sri Lanka; ^2^ Department of Agricultural Biology, Faculty of Agriculture University of Peradeniya Peradeniya Sri Lanka; ^3^ Department of Nursing, Faculty of Allied Health Sciences University of Peradeniya Peradeniya Sri Lanka; ^4^ Department of Internal Medicine Central Regional Referral Hospital Gelegphu Bhutan; ^5^ Department of Global Health and Development London School of Hygiene and Tropical Medicine London UK; ^6^ Department of Chemistry, Faculty of Science University of Peradeniya Peradeniya Sri Lanka; ^7^ Faculty of Dentistry University of Puthisastra Phnom Penh Cambodia; ^8^ School of Dentistry University of Rwanda Kigali Rwanda; ^9^ School of Health and Life Sciences Teesside University Middlesbrough UK; ^10^ Cephas Health Research Initiative Inc Ibadan Nigeria

**Keywords:** drug carriers, drug delivery systems, nanoparticles, Nanoplatform, nanotechnology, oral cancer

## Abstract

**Background and Aims:**

Cytotoxicity is a key disadvantage of using chemotherapeutic drugs to treat cancer. This can be overcome by encapsulating chemotherapeutic drugs in suitable carriers for targeted delivery, allowing them to be released only at the cancerous sites. Herein, we aim to review the recent scientific developments in the utilization of nanotechnology‐based drug delivery systems for treating oral malignancies that can lead to further improvements in clinical practice.

**Methods:**

A comprehensive literature search was conducted on PubMed, Google Scholar, ScienceDirect, and other notable databases to identify recent peer‐reviewed clinical trials, reviews, and research articles related to nanoplatforms and their applications in oral cancer treatment.

**Results:**

Nanoplatforms offer a revolutionary strategy to overcome the challenges associated with conventional oral cancer treatments, such as poor drug solubility, non‐specific targeting, and systemic toxicity. These nanoscale drug delivery systems encompass various formulations, including liposomes, polymeric nanoparticles, dendrimers, and hydrogels, which facilitate controlled release and targeted delivery of therapeutic agents to oral cancer sites. By exploiting the enhanced permeability and retention effect, Nanoplatforms accumulate preferentially in the tumor microenvironment, increasing drug concentration and minimizing damage to healthy tissues. Additionally, nanoplatforms can be engineered to carry multiple drugs or a combination of drugs and diagnostic agents, enabling personalized and precise treatment approaches.

**Conclusion:**

The utilization of nanoplatforms in oral cancer treatment holds significant promise in revolutionizing therapeutic strategies. Despite the promising results in preclinical studies, further research is required to evaluate the safety, efficacy, and long‐term effects of nanoformulations in clinical settings. If successfully translated into clinical practice, nanoplatform‐based therapies have the potential to improve patient outcomes, reduce side effects, and pave the way for more personalized and effective oral cancer treatments.

## Highlights

- Surgery, radiation, and chemotherapy are the solely available treatments for cancer.
- Oral cancer includes cancers of the mouth and the back of the throat.
- With selective targeting and distribution of these anticancer medications to tumor tissues, nanotechnology has traditionally been used in cancer therapy to enhance pharmacokinetics and lower the systemic toxicity of chemotherapy.
- Nanotechnology has the potential to overcome many shortcomings of chemotherapy, enable drugs to precisely target tumor cells, reduce unfavorable side effects on nearby healthy tissues, and thus pave the way to create cutting‐edge treatments for oral cancer.

## 1 INTRODUCTION

**Figure 1:**
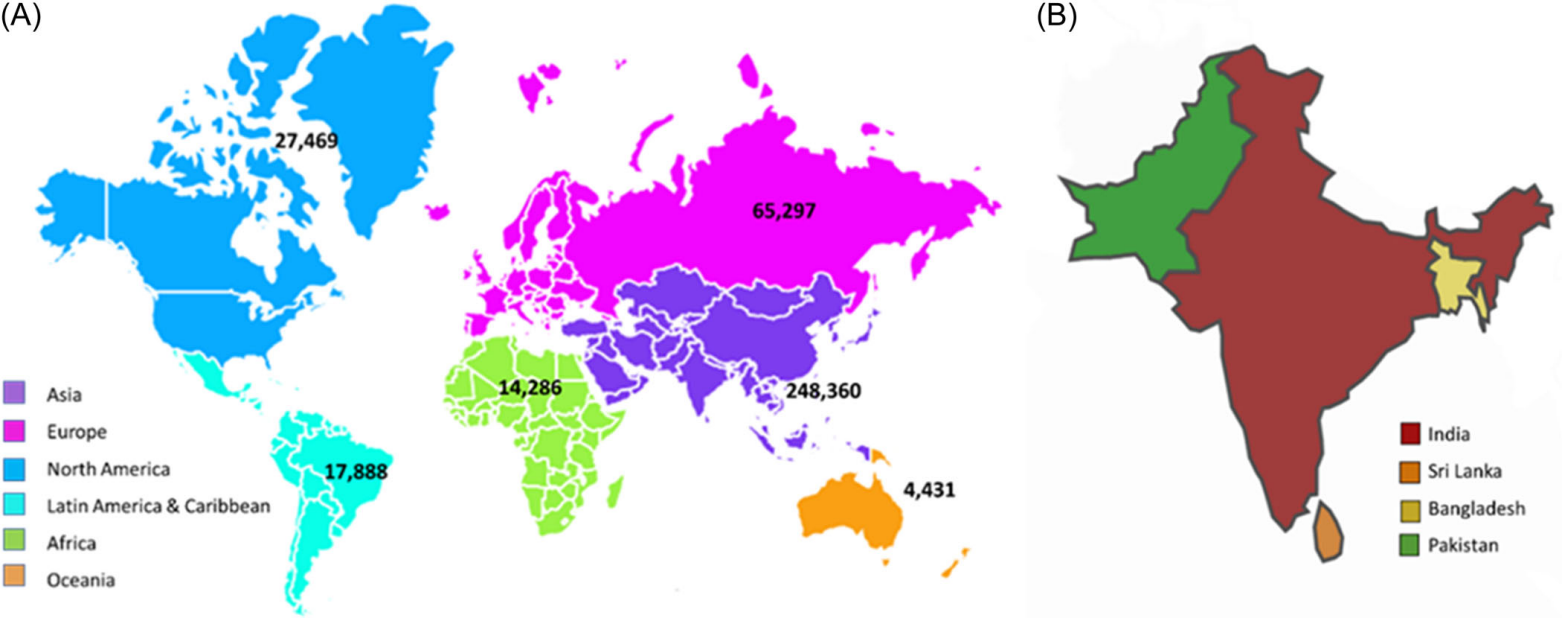
Lip and mouth cancer incidence in 2020. (A) The worldwide prevalence of lip and oral cancer of both genders is based on regions.^4^ (B) Areas having a greater prevalence of lip and mouth cancer.^5^

Oral squamous cell carcinoma (OSCC) is a common epithelial carcinoma with a complex origin that is affiliated with significant mortality and morbidity. OSCC is a malignant neoplasm that appears in the oral cavity and lips. Ninety percent of neoplasms in the oro‐facial region originate from squamous epithelial cells and are potentially fatal, disfiguring, and incapacitating. In most cases, OSCC arise from pre‐existing oral lesions referred to as oral potentially malignant disorders (OPMD).^1^, ^2^ Oral carcinoma is one of the top six cancer reported globally. In 2020, there were 377,713 new cases and 177,757 deaths related to cancers of the lip and mouth.^3^ South and Southeast Asia (e.g., India, Pakistan, Taiwan, and Sri Lanka), some parts of Eastern Europe, Western Europe, some regions of Latin America and the Caribbean, and Pacific regions have higher incidence rates for oral cancer (excluding lip) (Figure 1). Oral cancer is the most frequent type of cancer in these countries, which accounts for more than 25% of all new cancer cases.^6^ Oral cancer incidence rises with age, peaking in persons over 60 and among those under 40 years.^6^ Oral cancer can be influenced by various factors such as excessive tobacco consumption (both smoked and smokeless forms), betel nut chewing, alcohol, and chronic inflammation.^7^, ^8^ Human papillomavirus‐related (mostly HPV16) oral and oropharyngeal malignancies have become more common in young people in recent decades.^9^, ^10^, ^11^

In the realm of oncology, chemotherapy is the anticancer therapy that is most frequently used. Chemotherapeutic delivery can result in a variety of cutaneous modifications, from allergic reactions to infectious complications brought on by compromised immunity. The chemotherapeutic agents used in the treatment of oral cancers include cisplatin (DDP),^12^, ^13^ 5‐fluorouracil (5‐FU),^14^ docetaxel,^15^ paclitaxel,^16^ and methotrexate.^17^ Most of these oncological treatments are typically administered intravenously and are distributed to all tissues. Some physicochemical properties of anticancer drugs, such as poor water solubility, low bioavailability, adverse reactions with components of blood, and high toxicity, make oral delivery difficult.^18^ Nanoparticles (NPs) can transport medications trapped or encased in them for delivery to targeted sites in the body and release them only in the vicinity of cancerous sites.^19^ NP delivery also ensures the stability of the drug molecule before it is released at the intended site of action.^20^ Nanoplatforms can address the drawbacks related to conventional chemotherapy and allow precise targeting of tumor cells and minimize adverse effects on neighboring healthy tissues, allowing optimization of dose and bioavailability of the chemotherapeutic agent.^21^ Targeting, both passive and active, permits treatment while reducing the ill‐effects commonly associated with typical cancer treatments, such as hair loss and nausea.^22^ In this article, we will look at the latest breakthroughs in the use of nanotechnology in the treatment of oral malignancies.

## 2 ISSUES OF CONCERNS REGARDING CHEMOTHERAPY

A patient who undergoes surgery for a certain tumor is given specific postoperative instructions during typical cancer therapy, especially if the tumor is aggressive. Chemotherapy targets the body's remaining proliferating cells by periodically delivering a specific hazardous drug into the patient. This approach, however, has drawbacks, such as its lethal effect on healthy cells in the body. As a result, the medication provided to tumor cells kills normal cells, causing brain toxicity, bone marrow suppression, cardiomyopathy, and other issues.^23^

### 2.1 Nano etymology

The word “nano” comes from *nanos* in Greek, which also means “dwarf.” The one billionth part (10^−9^) of a unit was given the prefix nano at the 14th International Union of Pure and Applied Chemistry (IUPAC) meeting in 1947. In several domains of modern science, the prefix nano has become a prominent name to represent minuscule entities and processes in scientific literature. This category includes terminology such as nanoscience, nanotechnology, nanorobots, nanomagnets, nanoelectronics, nanoencapsulation, and others.^24^ In all of these examples, the prefix nano is used to designate “very small” entities or processes, most typically at the nanometer scale.^24^

### 2.2 Definitions

With an emphasis on the distinct characteristics of size‐dependent properties in solid‐state materials, the science field of nanoscience investigates the properties of material at the nanoscale.^25^ Nanotechnology is the scientific investigation of synthesis, engineering, and application of materials known as nanomaterials, which range in size from 1 to 100 nm.^26^ The American Physical Society meeting in 1959, where Nobel winner Richard Feynman delivered his well‐known lecture “There's Plenty of Room at the Bottom,” is frequently credited as the starting point for the development of concepts in nanoscience and nanotechnology.^27^ However, the usage of nanotechnology and nanomaterials has a considerably longer history.

**Figure 2:**
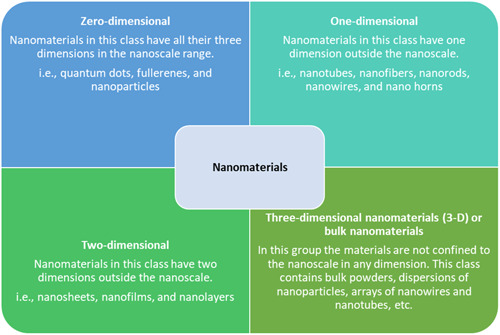
Dimensional classification of nanomaterials.

Nanomaterials have various applications in water treatment, catalysis, medicine, agriculture, energy storage, and more, due to their unique properties.^28^, ^29^, ^30^ Nanomaterials behave significantly different than larger‐scale materials due to surface effects and quantum phenomena.^31^ Nanomaterials have increased mechanical, thermal, magnetic, electrical, optical, and catalytic characteristics as a result of these variables.^24^ Nanomaterials are essential components of nanotechnology. They are described as materials with at least one nanoscale dimension, that is, smaller than 100 nm.^32^ Based on their dimensions, nanomaterials are divided into four types: zero‐dimensional nanomaterials (0‐D), one‐dimensional nanomaterials (1‐D), two‐dimensional nanomaterials (2‐D), and three‐dimensional nanomaterials (3‐D)^33^ (Figure 2).

The International Organization for Standardization defines NPs as nano‐objects with all exterior dimensions in the nanoscale and no substantial difference in the lengths of the nano‐object's longest and shortest axes. If the dimensions differ greatly (usually by more than three orders of magnitude), words such as nanofibers or nanoplates may be preferred to NPs. NPs come in a variety of shapes, sizes, and structures. They can have irregular shapes in addition to being spherical, cylindrical, conical, tubular, hollow core, and other configurations.^34^ NPs can range in size from 1 to 100 nm. The term “atom clusters” is used to describe NPs that are less than 1 nm in size. Crystalline NPs can be found in amorphous NPs with single or many crystals. NPs can be clumped together or loose.^35^ In the latter case, the layers are frequently: (a) The surface layer, which typically contains small molecules, metal ions, surfactants, or polymers. (b) Different molecules from those in the core layer make up the shell layer. (c) The core layer lies in the center of the structure.^36^ NPs can be classified as organic, carbon‐based, or inorganic depending on their composition.^34^

### 2.3 Organic NPs

**Figure 3:**
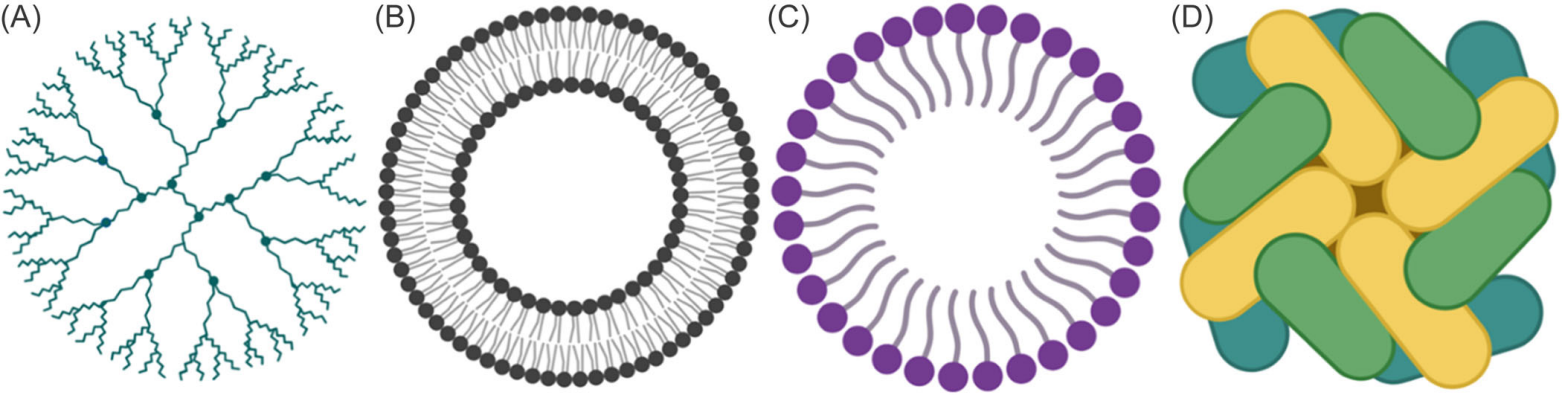
Organic nanoparticles (NPs) are classified into several types. (A) Dendrimers, (B) liposomes, (C) micelles, and (D) ferritin.

Organic NPs comprise of lipids, polymers, proteins, carbohydrates, and other organic materials.^37^ Dendrimers, liposomes, micelles, and protein complexes including ferritin are the most widely known in this category (Figure 3). These NPs are normally harmless, biodegradable, and can consist a void in its core, as in liposomes. Organic NPs are thermally and electromagnetic radiation sensitive.^34^ Additionally, they are frequently produced via noncovalent intermolecular interactions, which reduces their stability in nature and offers a mechanism to remove them from the human system.^38^ Composition, surface structure, stability, carrying capacity, and other factors all have an impact on the potential application sector of organic NPs. In biomedicine, organic NPs are now primarily used for the targeted drug administration as well as the treatment of cancer (Figure 3).^34^, ^39^

### 2.4 Carbon‐based NPs

**Figure 4:**
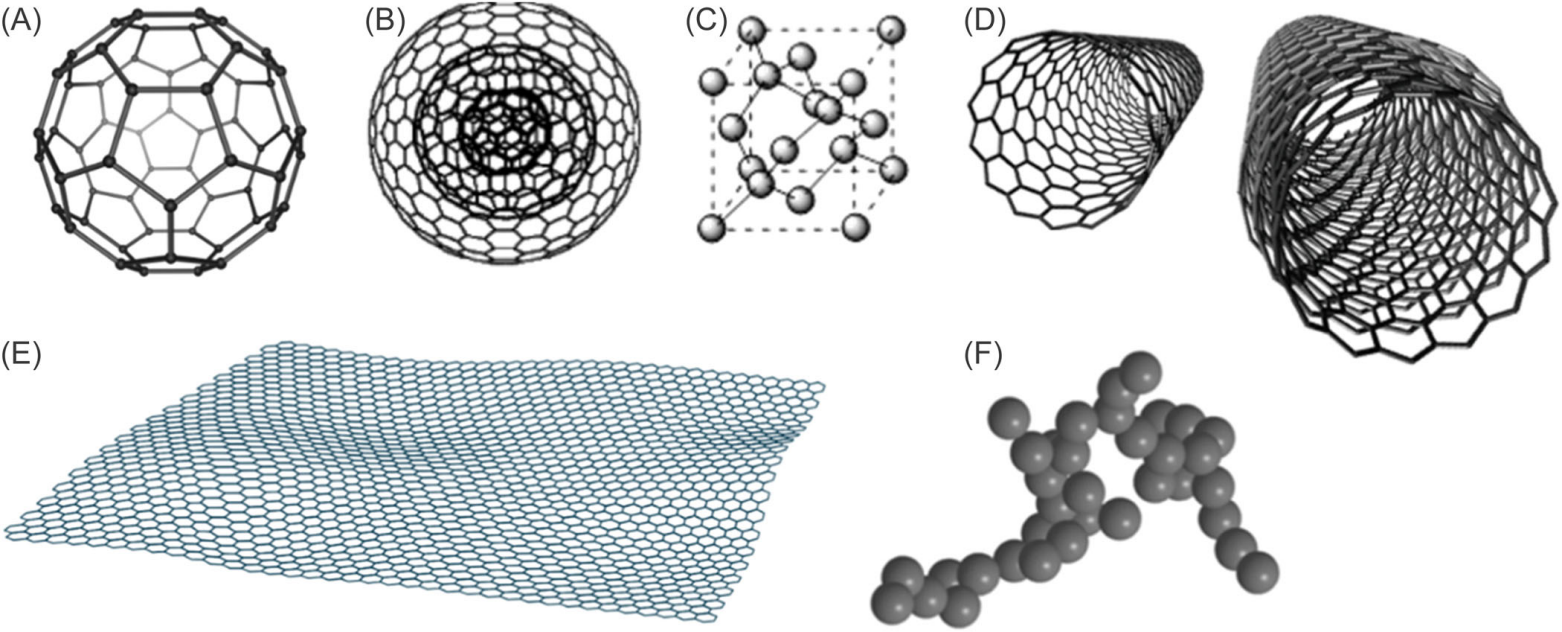
Examples of carbon‐based nanoparticles (NPs). (A) C60 fullerene, (B) carbon nano onions carbon, (C) nanodiamonds, (D) nanotubes, (E) graphene, and (F) carbon‐based quantum dots.

NPs formed completely of carbon atoms belong in this class.^34^ In terms of carbon‐based nanomaterials, fullerenes, carbon nanotubes, graphene and its derivatives, graphene oxide, nanodiamonds, and carbon‐based quantum dots are a few examples (Figure 4). Fullerenes are symmetrical carbon molecules having a closed‐cage structure. C60 fullerenes are made up of 60 carbon atoms organized like a soccer ball.^40^ The shape of grapes can be seen in aggregates of highly fused, spherical carbon black NPs.^41^ Carbon NPs with diameters smaller than 10 nm are used to create discrete, quasi‐spherical carbon quantum dots.^42^ Applications for carbon‐based NPs include the administration of drugs,^36^, ^43^ including drug delivery,^44^ energy storage,^45^ bioimaging,^46^ photovoltaic technology, and environmental sensing to track microbial ecology or identify microbial diseases. This is attributable to their distinct electrical conductivity, high strength, electron affinity and also thermal, absorption, and optical properties.^43^ More sophisticated structures are carbon nano onions and nanodiamonds. They have been utilized in tissue engineering and applications involving drug delivery due to the low toxicity and biocompatibility (Figure 4).^47^

### 2.5 Inorganic NPs

NPs in this category are not composed of neither carbon nor organic compounds. The most prevalent scenarios of this kind of material are semiconductor, metal, and ceramics. Metal NPs are formed exclusively from metal predecessors and can be mono‐, bi‐, or poly‐metallics.^48^, ^49^ Alloys or multiple layers (core‐shell) can be used to create bimetallic NPs.^49^ These NPs show distinct optical and electrical properties due to localized surface plasmon resonance characteristics.^36^ Additionally, several metal NPs exhibit unique thermal, magnetic, and biological characteristics.^34^ They are becoming more and more important materials because they can be utilized to make tiny electronic devices for a range of physical, chemical, biological, biomedical, and pharmaceutical reasons.^50^ For the development of cutting‐edge materials in the modern world, size‐, shape‐, and facet‐controlled metal NPs synthesis is essential.^51^

**Figure 5:**
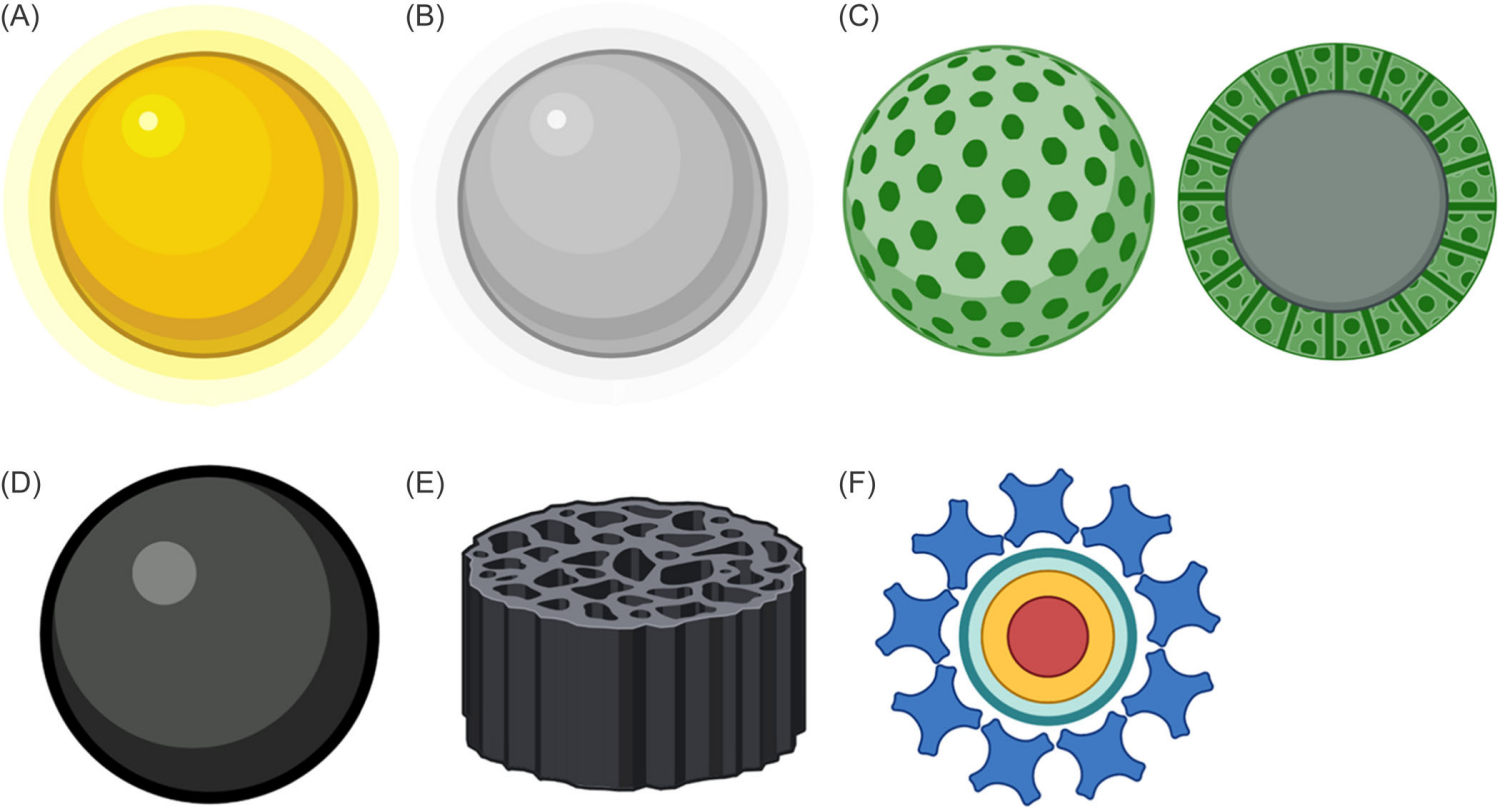
Several kinds of inorganic nanoparticles (NPs). (A) Gold NPs, (B) NPs, (C) mesoporous silica NPs, (D) Iron oxide NPs, (E) porous silicon NPs, (F) quantum dot nanocrystal.

Semiconductor NPs are made from materials with properties that are halfway between those of metals and nonmetals. When compared to bulk semiconductor materials, these NPs display considerable property changes with bandgap tuning and have discrete wide bandgaps.^36^ This implies that these NPs are essential for photocatalysis, optical technology, and electrical appliances.^52^ The inorganic solids known as ceramic NPs are composed of carbonates, carbides, phosphates, and metal and metalloid oxides such as calcium and titanium.^53^ The technique of heating and cooling substances repeatedly is frequently used to make them. They may be hollow, amorphous, polycrystalline, dense, or porous.^36^ Due to their outstanding stability and load capacity, ceramic NPs have many biological applications.^54^ Several other fields, such as optoelectronics, photonics, catalysis, and dye degradation additionally make use of them (Figure 5).^53^

### 2.6 Nanomedicine

**Figure 6:**
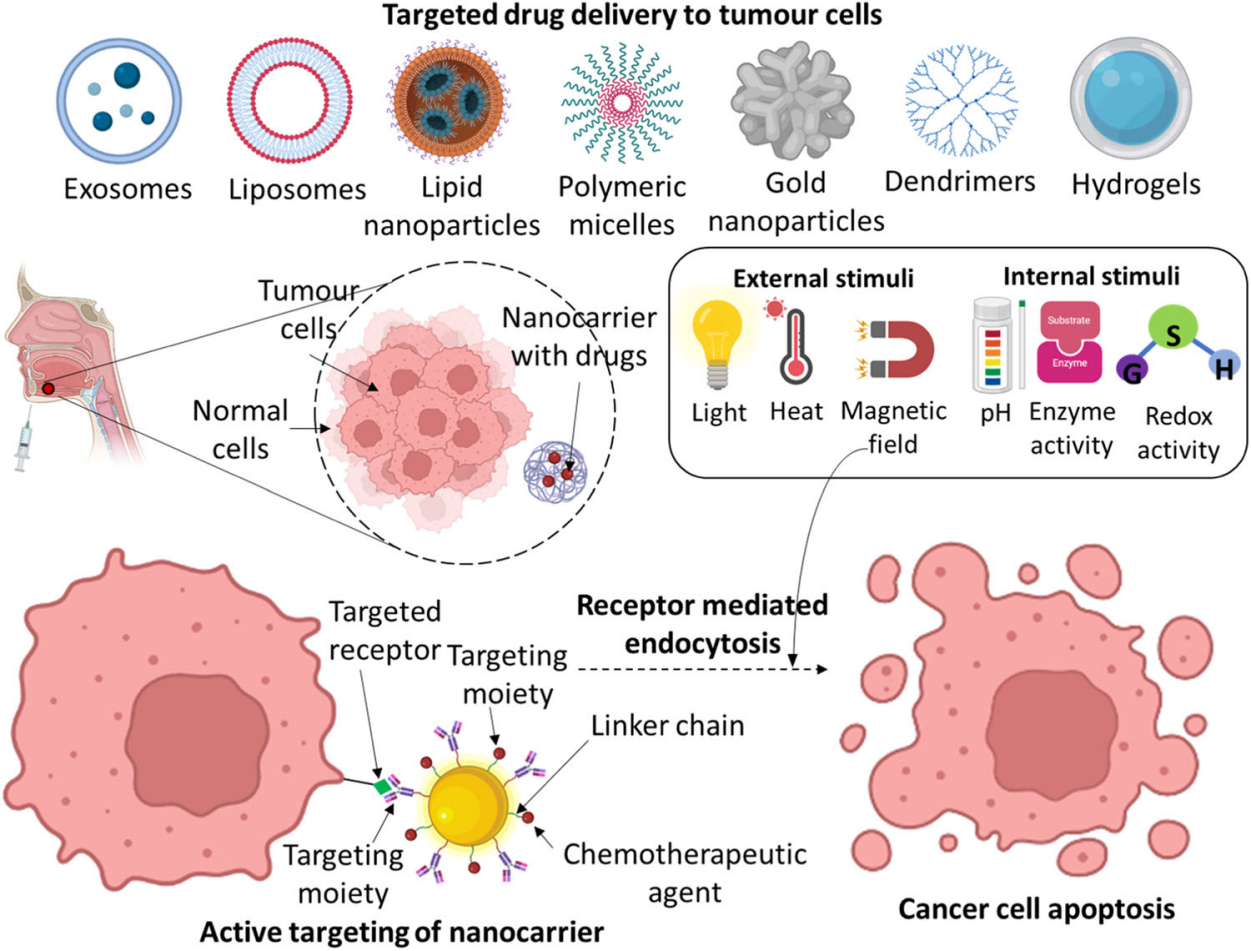
Schematic representation of a drug delivery system.

With a focus on creating tiny medical equipment to create an efficient healthcare system, nanomedicine is currently one of the most popular uses of nanotechnology. With the use of this strategy, we can better comprehend human physiology and fight against various devastating diseases like cancer and heart disease. The primary uses of nanomedicine are in image processing, disease diagnosis, tissue engineering, and the creation of more efficient, safe, and affordable drug delivery systems (DDS) to deliver drugs precisely to target sites, which can shorten the duration of treatment by reducing toxicities and off‐target effects.^55^ Additionally, by regulating the frequency, timing, and site of release, DDS is a formulation or instrument that facilitates the intake of a medicinal compound, improving both its efficacy and safety.^56^ Recently, notable progress has been made in the realm of delivery systems designed to transport therapeutic agents or naturally derived active substances to specific sites within the body, with the aim to treat a wide range of disorders.^57^ Several successful DDS developments have taken place in recent years; nevertheless, to successfully distribute drugs to their intended places, there are still several challenges that must be overcome and advanced technologies developed. As a result, nano‐DDS are currently being investigated to facilitate improved DDS (Figure 6).^58^

## 3 APPROACHES OF NPs IN ORAL CANCER TREATMENTS AND DETECTION

To precisely bind to the receptors on the overexpressed OSCC cell surface, polymeric NPs are utilized as ligand‐decorated NPs.^59^, ^60^ In addition, DDSs containing various medications, such as cisplatin and chrysin, are created as polyelectrolyte‐assembling multilayer NPs (MLNPs). According to Mehnath et al.,^61^ combinational polyelectrolyte‐assembling MLNPs exhibit an exceptional therapeutic impact when compared to single‐cisplatin‐loaded MLNPs. Also, specific NPs are designed to target cell signaling pathways in treating oral cancer. One such example is polyethene‐glycol‐polyethyl‐eneimine‐chlorin e6 NPs, where a Wnt‐1 siRNA is effectively transferred into the cytoplasm of OSCC. Inhibiting the Wnt/‐catenin signaling pathway, which is crucial for the epithelial‐mesenchymal transition, is a side effect of photodynamic treatment.^62^

Furthermore, NPs can be utilized to identify oral cancer early.^63^ The most accurate way to diagnose oral cancer is through a biopsy,^64^ but this procedure is invasive and uncomfortable. Nano‐based imaging techniques include Nano‐MRI,^65^ optical coherence tomography, photoacoustic imaging, SPR extinction, surface‐enhanced Raman spectroscopy (SERS), diffusion reflection imaging, quantum dot‐based imaging, and nano‐based ultrasensitive biomarker detection are upcoming nano‐based oral cancer detection methods.^66^ Nano‐based cancer detection methods enable the visualization of cytologic and morphologic deviations of cancer cells, including nuclear size, epithelial thickness, and blood flow. Also, it can differentiate cancers and premalignant lesions. There are surgical benefits of nano‐based detection methods, which help to determine the tumor margins and lymphatic metastasis.^67^ Despite the potential of NP‐based therapies for oral cancer, some challenges must be addressed.^68^ One major challenge is the development of efficient and safe methods to deliver NPs to the tumor site.^69^ Another challenge is the potential toxicity of NPs, as some can cause inflammation and immune reactions in the body.^70^

The detection and diagnosis of patients with oral cancer using nanotechnology‐based approaches is becoming more noninvasive than conventional cancer detection methods. Chemotherapy resistance is another trending issue in patients with cancer; nanotechnology has some therapeutic advances in patients with chemotherapy resistance. Nano‐particle‐based DDSs act on several mechanisms to overcome chemotherapy resistance. Inhibition or bypass of efflux transporters, activation of apoptosis, inhibition of efflux transporters while activating apoptosis, and targeting hypoxia are the main mechanisms that interfere with chemotherapy resistance in patients with cancer.^71^ Cancer immunotherapy is an effective treatment strategy in patients with oral cancer. NPs have a significant role in cancer immunotherapy. This approach allows the incorporation of NPs with immunomodulatory agents by activating immune cells and regulating the tumor microenvironment to enhance antitumor immunity.^72^ Nano‐vaccines, artificial antigen‐presenting cells are oncologic advances in nano immunotherapy. Nano vaccines carry tumor‐associated antigens and activate cytotoxic T cells. Artificial antigen‐presenting cells joined with MHC antigen complexes, and supportive stimulatory molecules result in T‐cell activation.^73^

### 3.1 Gold NPs

Due to their nontoxicity for healthy tissues or cells, higher resistance to organic solvents, and improved bioavailability compared to free drug, inorganic NPs have been utilized in therapies on a broad scale.^74^ A range of inorganic minerals, including copper, iron, titanium, silver, gold, and silica, are used in NP form, especially for cancer treatments.^75^ Because of its strong metallic contacts, Gold is considered to be one of the most stable metals.^76^ Sulfur and Nitrogen‐containing groups can form stable chemical interactions with gold NPs (AuNPs), allowing organic ligands or polymers to be attached for simple surface modification.^77^ AuNPs are extensively used in cancer therapy because to their remarkable properties including SERS, surface plasma resonance (SPR), controlled synthesis, surface morphological flexibility, biological safety, and stability.^78^ Photothermal treatment (PTT) uses AuNPs to eliminate cancer cells through the conversion of light energy into thermal energy in the desired areas of the mucous membranes and skin while leaving healthy tissues undisturbed.^79^, ^80^

It is crucial for effective treatment to diagnose oral cancer. In recent years, several studies have been developed that assist in the early detection of oral cancer employing SERS, optical coherence tomography, also known as OCT, and diffuse reflectance (DR) spectroscopy supplemented with AuNPs. SERS can be utilized as an optical imaging technique through tracking certain molecules. The primary advantage of SERS imaging is its high spatial resolution, which can reach 0.5 m in the visible range, allowing for high‐resolution specimen mapping. An additional advantage is the capacity to carry out multiple tests for several analytes in an identical sample.^81^ Chakraborty et al. invented a noninvasive enzyme‐linked immunosorbent test (ELISA) based on AuNPs to detect osteopontin in oral cancer.^82^ This modified ELISA has a wide linear detection range (0.31–20 ng/mL), high reproducibility, and specificity for the tested interference in saliva.^82^ In comparison to conventional ELISA kits (detection limit: 0.14 ng/mL), oral cancer detection with gold nanorod‐ or gold nanosphere‐based ELISA kits (detection limit: 0.02 ng/mL) is more accurate.^82^ Furthermore, Liu et al.^83^ proposed a rapid, noninvasive, extremely sensitive cancer screening approach that combines SER scattering technology with exfoliated cytology. A plasma gold nanorod‐based SER scattering matrix with filter paper adsorbed was created for cancer screening. Raman spectroscopy depicts cancer molecular alterations, displaying excellent specificity of cell and tissue biochemical components. Different and repeatable SERS spectra can be produced from normal cells and cancer cells due to changes in certain biomolecules in cancer cells. The detection of exfoliated cells in both healthy and cancerous tissues can be done with good sensitivity and specificity using a diagnostic strategy based on spectral value ratios.^83^

OCT was initially described in 1991 as a method for producing light‐interfering, two‐dimensional cross‐sectional pictures. Since it detects reflected light from tissue rather than sound, it is frequently referred to as an optical analog of ultrasound.^84^ OCT imaging technique has cross‐sectional and 3D image resolutions of a few microns and penetration depths of 1–2 mm.^85^ Contrast compounds can raise the quality of OCT in situ images to that of biopsy images, eliminating the need for tissue removal, sectioning, and staining.^86^ It is easier to distinguish between normal tissue and malignant tissue when contrast chemicals are added to the OCT system. AuNPs are currently the most popular contrast agent because they have the best biocompatibility, are simple to make, can be used either systemically or locally, and are all effective.^87^ With the help of acid‐degradable gold nanoclusters,^88^ Kim et al. employed OCT to locate tumor tissue that was somewhat acidic.^87^ They created a new platform for converting AuNPs into water‐soluble microneedles.^87^ Then, in vivo delivery of AuNPs to the hamster's oral tissue was carefully regulated.^87^ The findings demonstrated a 150% increase in resolution when utilizing AuNPs for OCT detection.^87^ However, the penetration depth is just a few millimeters^89^ due to absorption and scattering restrictions, necessitating more profound investigation and advancements for better clinical application

DR spectroscopy, a brand‐new optical method for biological imaging, has been developed recently. By assessing intrinsic light absorption and scattering characteristics at various wavelengths, tissue features can be detected using DR.^90^ By exposing the tissue to the chosen spectrum, the optical fingerprint of the tissue is produced. Specific quantitative biochemical and morphological data are represented by this fingerprint.^90^ The benefits of DR include its comprehensiveness, simplicity, and great diagnostic potential. Additionally, a plethora of information about biochemical and ultrastructural changes can be found in the DR spectra of tissues.^91^ Tissue arrangement, intravascular oxygenation, metabolic rate, and blood vessels have an impact on the observed DR spectrum features.^90^ As a result, DR can provide in‐depth information about the biological structure of tissues beneath layers, potentially distinguishing between malignant and healthy tissue. Because of their unique optical properties, AuNPs can improve tissue signal absorption and dispersion. Additionally, by concentrating on aberrant regions, DR signals' specificity can be improved by AuNPs, which can also discriminate between sections of normal and diseased tissue.^92^ Although AuNPs‐based detection methods offer a lot of potential for early oral cancer diagnosis, in vivo testing is still necessary to determine how accurate and practical they are. However. OSCC and severely dysplastic lesions remain difficult to diagnose and detect.^78^

### 3.2 Silver NPs

AgNPs are silver NPs with diameters ranging from 1 to 100 nm with distinctive features such as electrical, optical, and magnetic capabilities with broad use.^93^ When metallic silver ions get into contact with a reducing substance, they ionize and become active. Ionic silver is a form of active silver that attaches to bacterium cell walls, causing significant structural alterations in cell shape. AgNPs cause RNA and DNA replication denaturation, which results in cell death.^94^ Because of its bactericidal potential at low concentrations, silver is also known as oligodynamic. As a result, it has been widely used in medical products.^95^ After being taken up by endocytosis‐related pathways, AgNPs are gathered in endosomes and guided to lysosomal fusion. The acidic lysosomal environment increases the release of silver ions from AgNPs, which disrupts cellular homeostasis and, depending on the biological characteristic of the targeted cell, leads to apoptotic cell death.^96^ This is known as the “Trojan‐horse” mechanism, and it means that AgNPs' cytotoxicity develops only after they have been taken up by cells.^97^

**Figure 7:**
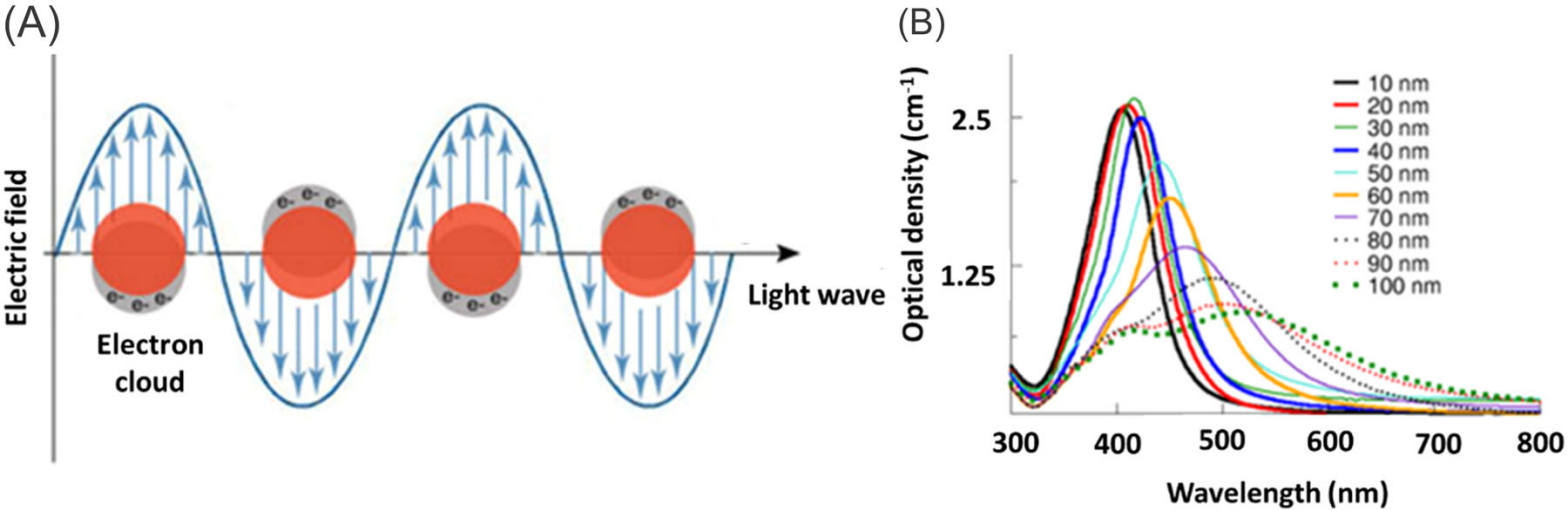
(A) Interactions of surface plasmons with the incident light wave^98^ and (B) the surface plasmon resonance spectra of gold nanoparticles (NPs) as a function of size^99^ (reproduced with permission).

Due to the carefully controlled optical properties, inorganic NPs made of noble metals, such as gold, have found extensive usage in diagnostic and imaging procedures. Particularly AgNPs and AuNPs have a SPR effect (Figure 6). NPs have many of their constituent atoms on the surface with metal ions embedded in a sea of electrons known as plasmons. The metal quantum dots have many surface plasmons vibrating at a particular frequency. This frequency depends on the number of surface plasmons and the size of the NPs. These wavelengths fall within the range of the visible light spectrum, 400–750 nm. When the frequency of the incident light matches that of the surface plasmon vibrations, it is absorbed giving rise to complementary color (Figure 7). Therefore, the size‐ and shape‐dependent SPR effect of AuNPs can be used to stain diseased organs in different colors. This feature of NPs can be applied in imaging for cancer detection. Surface plasmon vibrations can be tuned to occur in the near‐infrared radiation (NIR) generating heat. Such NPs can be utilized as photo‐thermal agents in medical settings. For the treatment of advanced and recurrent OSCC, photodynamic therapy can be used to improve the penetration of therapeutic agents into tissues.^100^

### 3.3 Liposomes

Lipid‐based NPs (LBNPs), such as liposomes, solid lipid NPs (SLN), and nanostructured lipid carriers (NLC), have garnered a lot of interest in the production of drugs for the treatment of cancer. These NPs may carry both hydrophobic and hydrophilic molecules, have very low or no toxic effects, and can prolong the duration of therapeutic activity by having a prolonged half‐life and controlled drug release.^101^, ^102^ Using gangliosides or polyethene glycol (PEG), lipid nanosystems can be chemically altered to avoid immune system detection or to increase medication solubility. Furthermore, they can be coupled with antibodies that recognize tumor cells or receptors (like folic acid) to boost drug release in an acidic environment. They can also be manufactured in pH‐sensitive formulations.^103^

**Figure 8:**
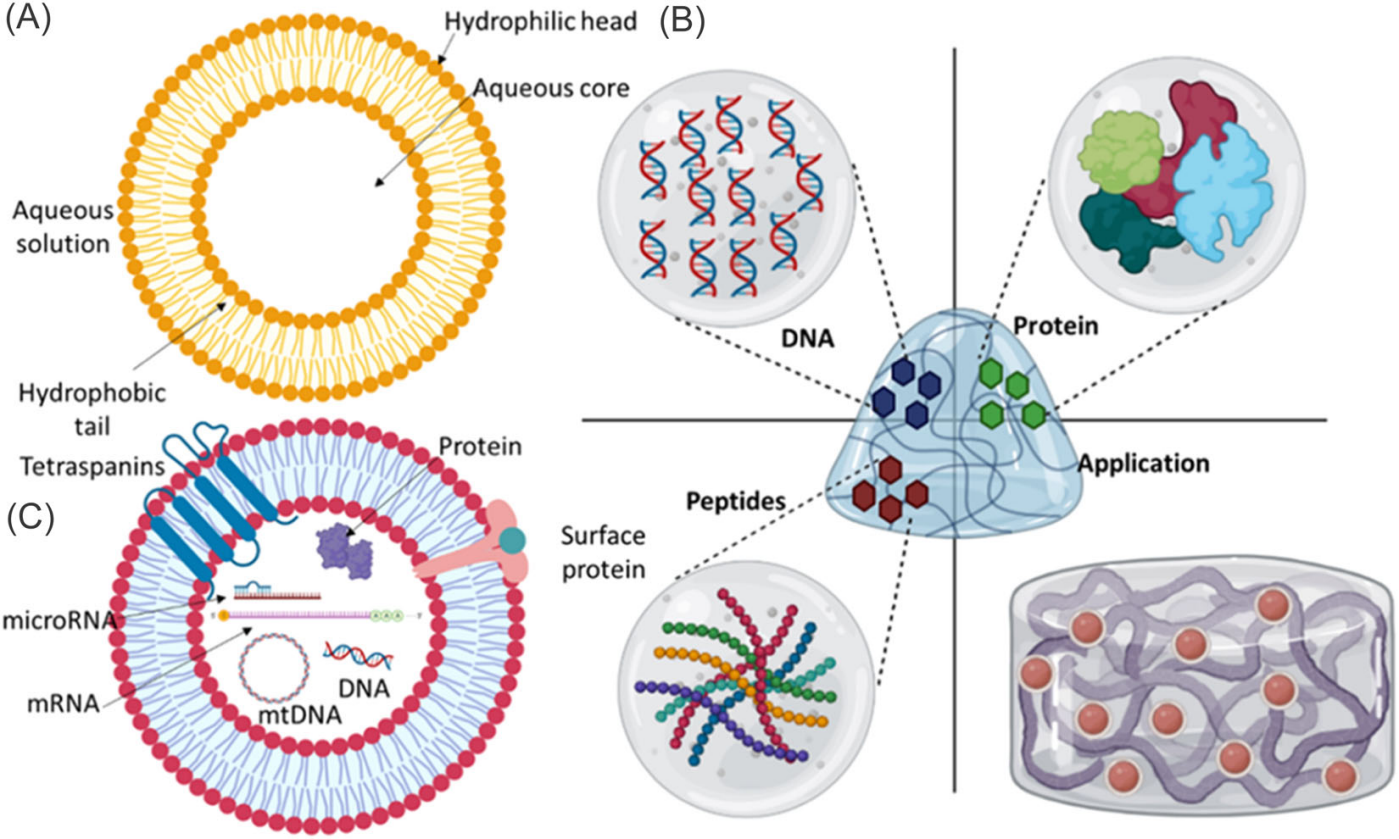
T typical structural arrangements of (A) liposome with aqueous core where polar drugs can be encapsulated and lipid bilayer arrangement where nonpolar drugs can be trapped,^105^ (B) hydrogel containing DNA, peptides, and proteins and its applications,^106^ (C) exosome.^107^

Liposomes are tiny vesicles with an aqueous center and one or more phospholipid bilayers surrounding them. The size of liposomes varies from 25 nm to several microns. They can be made from both natural and synthetic phospholipids and cholesterol. Hydrophobic pharmaceuticals are incorporated into the lipid bilayers, whereas hydrophilic medications are restricted to the hydrophilic core (Figure 8).^104^ When liposomes enter the human body, the mononuclear phagocytic cells immediately recognize them as foreign materials and take them up. For administering anticancer medications to the tumor cell, liposomes offer ideal carriers. Liposomes can thereby increase the effectiveness of anticancer medications and minimize their adverse effects.^108^ There are various ways that liposomes engage with the cell surface. Lipid exchange is the first and contributes to lipid molecule exchange between cell membranes and liposomes. The encapsulated material inside the liposome diffuses over the cell membrane in the second case, which is adsorption. Finally, liposomes could fuse with cell membranes to convey the material they have been encapsulating.^109^

Liposomes have long been employed in cancer research. Traditionally, radioactive tagging with a phospholipid bilayer or hydrophilic core has been accomplished through linking a radionuclide to an anchoring molecule. In comparison to 18F‐FDG, Mahakian et al. suggested that 64Cu liposomes might be able to identify malignancies at an early stage.^110^ Liposomes are commonly employed in anticancer therapy to improve efficacy and decrease negative effects. Polyethylene glycol (PEG), a synthesized biocompatible polymer, can be coated to the surface of the liposome to impede its clearance by the reticuloendothelial system and boost blood flow for a short period of time. Adding doxorubicin (DOX) to liposomes to generate anticancer drug formulations has been the subject of numerous investigations. El‐Hamid et al. hypothesized that PEGylated liposomal DOX increased apoptosis more than free DOX did in CAL‐27 cells.^111^ Despite being safe, prolonged PLD usage in some patients has been associated with precancerous or OSCC lesions.^112^, ^113^ Curcumin, paclitaxel, carboplatin, and cisplatin are additional drugs found in liposomes that are more successful at inducing the death of cancer cells.^114^ It was revealed that combining two or more anticancer medications with vitamins improved chemotherapeutic treatment efficacy. DOX and resveratrol encapsulated liposomal formulations, for example, have been examined in oral cancer, and when evaluated in vitro, the therapeutic combination showed greater efficacy in the treatment of oral cancer.^115^

Gene therapy formulations based on liposomes, meanwhile, show a lot of potential for treating oral cancer. Due to their simplicity in delivering therapeutic genes to target cells, liposomes are a possible replacement for viral vectors.^116^ To prevent tumor cell proliferation and negatively influence growth, Figueiredo et al. created liposomes for the delivery of p12. Studies conducted in vitro on resistant mouse squamous cell carcinoma VII (SCC‐VII) cells showed that this liposome composition was more damaging to cancer cells than naked DNA or other nonviral formulations.^117^ Liposomes have also been investigated for use as radionuclide carriers in tumor radiotherapy. After convection‐enhanced administration, loading 186Re into liposomes can successfully treat oral cancer with little side effects.^118^ Boron neutron capture treatment (BNCT), a tumor‐targeting therapeutic approach based on the preferentially selective uptake of 10B target species by tumor cells and neutron irradiation, has attracted the attention of researchers interested in effective tumor cell eradication. Using a cheek pouch cancer model in hamsters, research was conducted to study a boron‐rich liposomal system by Heber et al.^119^ Liposomes were used to deliver 10B drugs to tumor tissue only before neutron radiation. The reaction between neutrons and 10B atoms dramatically inhibited the development of tumor cells by producing short‐range particles.^119^

### 3.4 Hydrogels

Wichterle and Lim^120^ were the first to introduce the term “hydrogel” to describe a three‐dimensional (3D) framework built of hydrophilic polymers. The popularity of hydrogels is a result of four properties: biocompatibility, biodegradability, drug‐loading capacity, and controlled drug release (Figure 8). The hydrogel's biocompatibility suggests that it doesn't manifestly harm the body after being implanted and that it doesn't stimulate the immune system. Furthermore, many natural and synthetic polymers used to make hydrogels for cancer treatment are biodegradable, that is, chitosan (CTS)^121^ hyaluronic acid (HA),^122^ alginate (ALG),^123^ polyesters,^124^ and polyphosphazene.^125^

Using a syringe or catheter, hydrogels can be injected into the body.^126^ When chemotherapy drugs are injected to a tumor directly, the bloodstream rapidly eliminates them, therefore tumor eradication cannot occur since the chemicals cannot accumulate around the tumor tissue for a prolonged time period.^127^ To sustain a high drug dose in the tumor site for an extended period, and to reduce side effects and discomforts associated with frequent injections, a common approach involves encapsulating chemotherapy drugs in a hydrogel before implanting near the tumor. Moreover, the growing number of trials for in situ chemotherapy drug delivery is attributed to the widespread utilization of self‐healing hydrogels in tissue engineering and regeneration, such as in the context of wound healing. These hydrogels have gained popularity recently due to its biocompatibility and adhesive ability to surrounding tissues.^128^, ^129^, ^130^ Further, when compared to single‐drug chemotherapy, combining two or more chemotherapeutic drugs can reduce drug dosage, lessen severe side effects, and increase treatment effectiveness.^131^

Because they offer protection and are simple to inject, hydrogels are an excellent drug loading carrier. Kim et al. demonstrated a hydrogel‐based drug carrier for the continuous administration of DOX and 5‐Fu, two chemotherapeutic drugs, a maximum of 18 days drug delivery.^132^ 5‐Fu‐loaded pluronic hydrogel (5‐Fu‐HP) or 5‐Fu‐loaded diblock copolymer hydrogel (5‐Fu‐HC) were combined with DOX microcapsules which displayed sufficient fluidity for direct injection to the tumor. Inside the body, the hydrogel undergoes gelation at body temperature. Relative to microcapsules, the hydrogel demonstrated a longer sustained drug release period without transiently high concentrations, hence lowering hazardous drug levels. The controlled delivery of the drug offered by this hydrogel‐based drug delivery depot might be prolonged if the polymer structure was changed.^133^

### 3.5 Exosomes

A variety of cells release exosomes into the extracellular space, including dendritic cells, macrophages, mesenchymal stem cells, endothelium, and epithelial cells. Exosomes are membrane‐bound vesicles with diameters between 40 and 100 nm.^134^ Exosomes are naturally occurring NPs with a variety of biological and therapeutic uses. They are released when the multivesicular body fuses with the cell membrane. Exosomes, which can contain a variety of biomolecules, are essential for intercellular communication (Figure 3).^135^ They are reliable for transporting drugs with precise targeting ability since they can adhere to the plasma membrane via adhesion proteins and ligands.^134^ Utilizing them as a delivery system for chemotherapy drugs including curcumin, DOX, and paclitaxel (Taxol) could mitigate their negative effects while boosting their therapeutic potency (Figure 8).^134^

### 3.6 Dendrimers

Due to their many functional groups and rigid molecular structure, dendrimers are well‐defined synthetic macromolecules.^136^ Dendrimers are appealing oral cancer diagnostic techniques. To detect oral cancer biomarkers such interleukin‐8 RNA, interleukin‐8 protein, and interleukin‐1 protein, Wei et al. created DNA‐dendrimer and polypyrrole (DDPpy) sensors with greater specificity and affinity.^137^ Diagnostic imaging and anticancer treatment both rely significantly on dendritic macromolecules. Due to their advantages, these clearly defined materials are the latest form of macromolecular nano‐scale delivery systems.^138^ Dendritic macromolecules typically grow exponentially in diameter and adopt a more globular shape as dendrimer production increases. Therefore, dendrimers have become an ideal delivery vehicle candidate for research on how polymer size, charge, and composition affect biologically significant properties like lipid bilayer interactions, cytotoxicity, internalization, blood plasma retention time, biodistribution, and filtration.^139^

The most promising potential of dendrimers is the execution of regulated and targeted medication distribution, which is relevant to the field of nanomedicine. One of the most fundamental challenges facing modern medicine is enhancing the pharmacokinetic properties of cancer medications.^140^ Drugs that have been conjugated with polymers have a more prolonged half‐life, higher stability, more water solubility, and less immunogenic and antigenic potential.^141^ Passive targeting and the subsequent selective accumulation of macromolecules in tumor tissue are made possible by the pathophysiologies of tumors, such as excessive angiogenesis leading to hypervascularization, increased permeability of tumor vasculature, and restricted lymphatic outflow. This phenomenon is known as “enhanced permeation and retention” (EPR).^142^ The drug‐dendrimer conjugates are extremely soluble, exhibit little systemic toxicity, and accumulate specifically in solid tumors. It has been suggested that dendrimers can include medicinal substances, genetic materials, targeting agents, and colors by encapsulation, complexation, and conjugation.^140^

**Figure 9:**
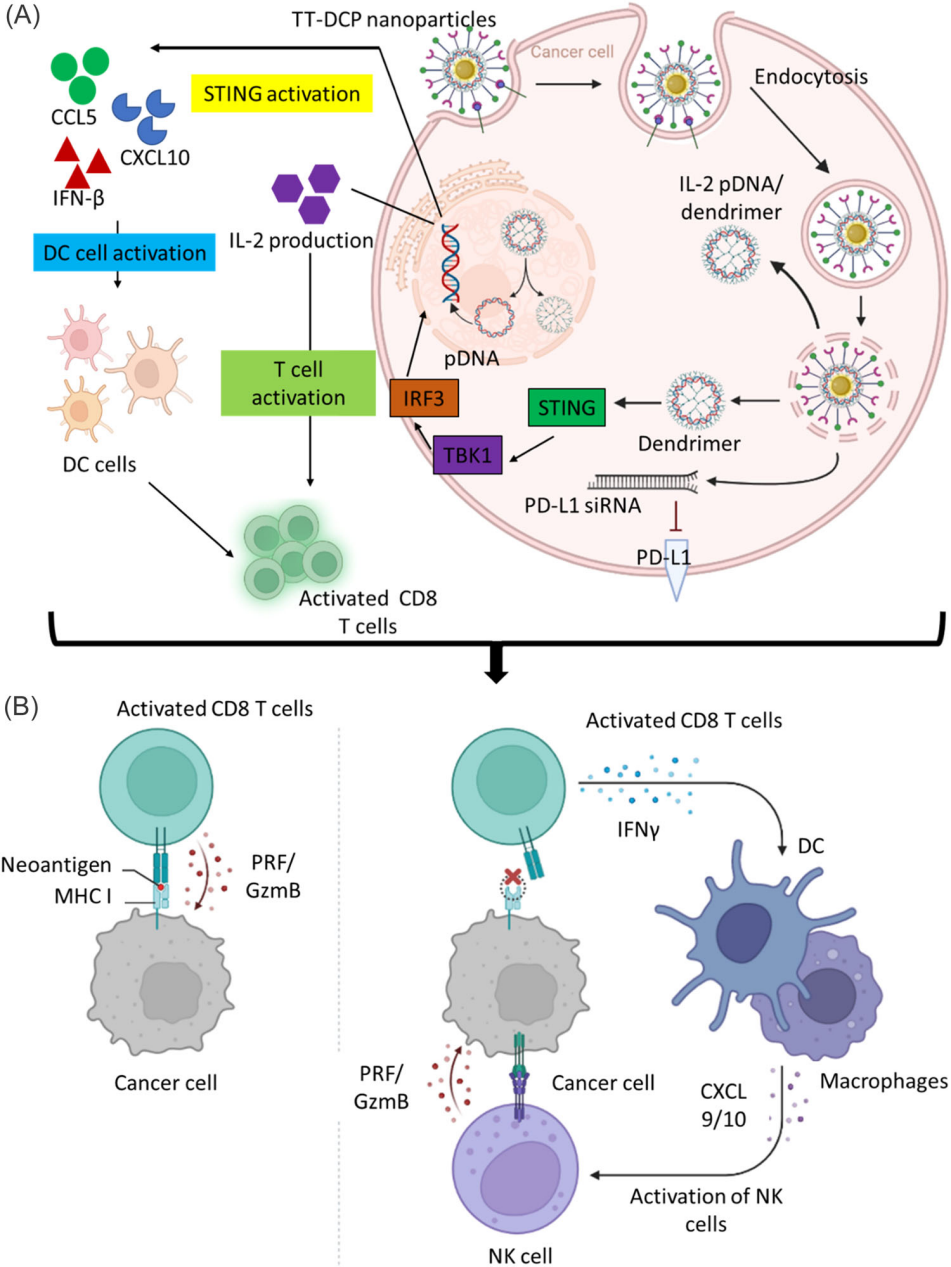
Schematic representation of the mechanism of dendrimer‐based immunotherapy (A) immunogene therapy by lipid‐dendrimer nanoparticles (NPs) containing siRNA against the immune checkpoint PD‐L1 and pDNA encoding the immunostimulating cytokine interleukin‐2, (B) CD8 T cell‐based cancer cell apoptosis, (C) NK cells‐based cancer cell apoptosis.

The underlying promise that discovering disease molecular pathways and the complete human genome sequence will lead to the development of safer, more effective medications and transform how we treat patients has not yet been realized. Genetic therapies will, however, undoubtedly make a significant contribution to the human therapeutic arsenal once some of the fundamental obstacles, such as focused and effective delivery, have been addressed.^143^ The delivery of DNA strands to a cell's essential components is fraught with difficulties. To carry genes into cells using dendrimers without compromising DNA integrity or deactivating them, research is currently being done in this area. The dendrimer/DNA complexes were encapsulated in a water‐soluble polymer and either placed on or positioned between functional polymer films with a fast breakdown rate to assist in gene transfection. For substrate‐mediated gene delivery employing PAMAM dendrimer/DNA complexes, this method encapsulates functional biodegradable polymer films. The fast‐degrading functional polymer offers a strong potential for targeted transfection, according to research.^144^ Dendrimers are able to combine therapeutic drugs with alternative targeted drugs on a single carrier device. Owing to the discovery of different alternative targets using dendrimer‐based technologies (Figure 9),^145^ future therapies (receptor targeting and therapeutic use) may be tailored based on the genetics of cancer features.

### 3.7 Magnetic nanoparticles (MNPs)

**Figure 10:**
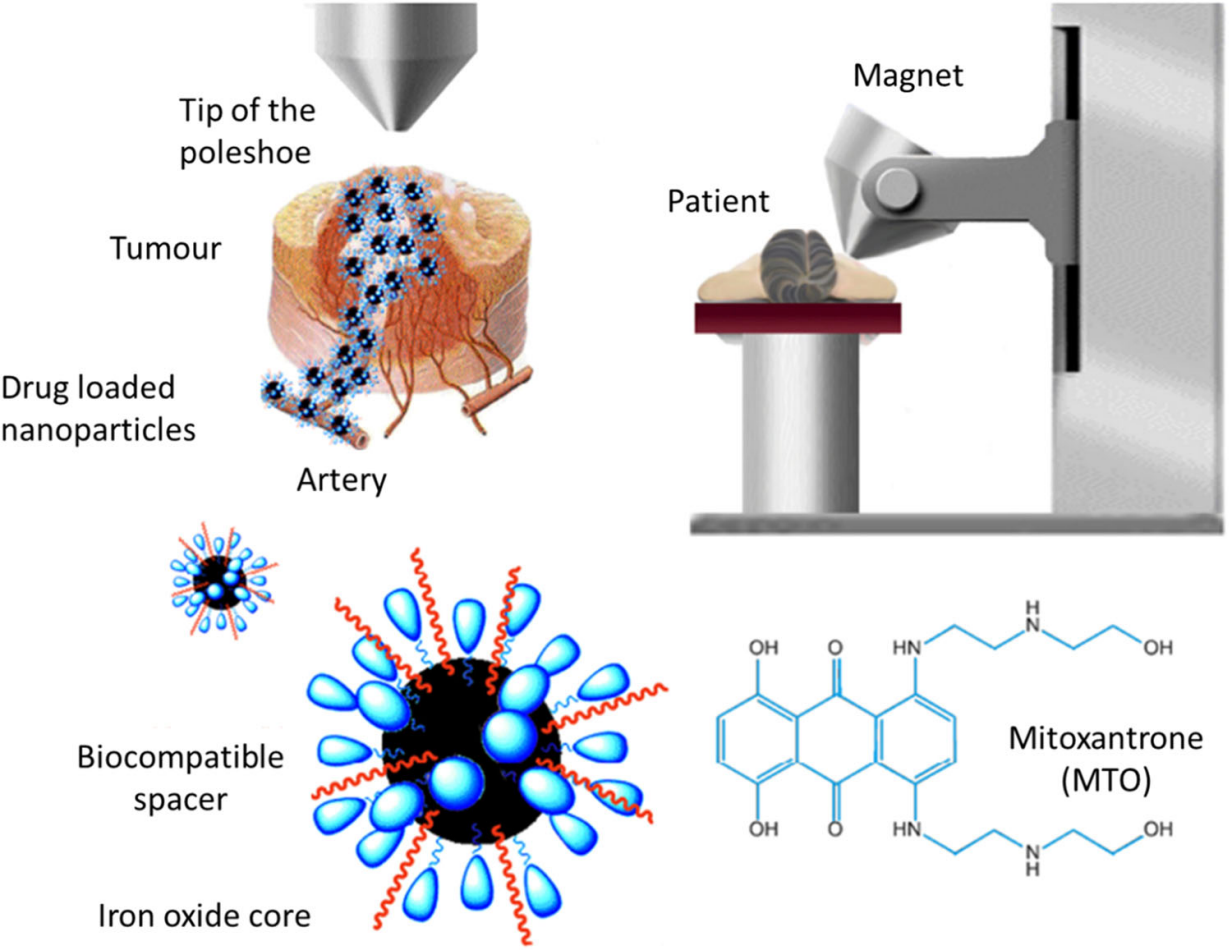
Schematic illustration of magnetic targeting of anticancer drug mitoxantrone (MTO) to cancerous sites. Reproduced with permission from Chimisso et al.^106^

Differently shaped and altered MNPs have outstanding magnetic properties as well as strong stability, biocompatibility, and biodegradability. Oral cancer is one of the numerous cancers that can benefit from the use of magnetic resonance imaging (MRI), DDSs, and hyperthermic treatment. MNPs can be made by coprecipitation, thermal decomposition, microemulsion, hydrothermal, sol‐gel, combustion, and polyol syntheses, among other techniques.^146^ These particles are made of iron oxide and can easily be manipulated using a magnetic field. Iron oxide NPs can be synthesized easily in different crystallographic phases such as magnetite, hematite, maghemite, and so on and all of which are magnetic NPs. This allows for the precise targeting and delivery of therapeutic agents to specific body areas.^147^ Fig. 1 in Janko et al.,^148^ reproduced (with permission) as Figure 10, shows how superparamagnetic iron oxide NPs (SPION) coated with lauric acid and loaded with mitoxantrone (SPIONMTO) are delivered to cancers in a targeted manner using an exterior magnetic field applied to the patient. Furthermore, MNPs can be heated using radiofrequency waves, which can aid to kill cancer cells and trigger the immune system to fight the remaining cancer cells.

MNPs are the most promising targeted DDS because they can assemble drugs specifically for the tumor site while being governed by a foreign magnetic field. However, MNPs' nonporous structure makes it challenging to apply drug carriers. To solve this issue, Zhang et al. proposed a solvothermal MNP system in which the outermost layer of MNP was modified with polyacrylic acid (PAA) to increase the transferring amounts of bleomycin (BLM). Under the influence of a magnetic field, BLM‐MNPs continuously accumulated in tumor tissue and, by progressively releasing BLM locally, inhibited the growth of the tumors.^149^ Because RNA interference (RNAi) contributes in the process of gene silencing in eukaryotes and can be activated by small interfering RNA (siRNA) and microRNA (miRNA), a recent research investigation on NP‐based gene therapy has shown promise for the treatment of cancer. Thus, MNPs might be created to target these genes. Inhibiting both B‐cell lymphoma‐2 (BCL2) and Baculoviral IAP repeat‐containing 5 (BIRC5) causes apoptosis. Jin et al. used Fe_3_O_4_ NPs to develop a siRNA delivery method for BCL2 and BIRC5. A positive charge was provided by MNP coated with polyethyleneimine (PEI) after cellular uptake, which is required for siRNA capture and gene silencing.^150^

Miao et al. utilized PEI‐modified Fe3O4 NPs driven by the hTERT tumor‐specific promoter to induce apoptosis by targeting the human‐TRAIL gene.^151^ Hyperthermia is more toxic to cancer cells than to healthy cells. PTT can cause tumor cell necrosis and degeneration in addition to inhibiting tumor cell proliferation. Gold NP‐mediated PTT can destroy oral carcinoma cells, but this technique is more usually used to treat superficial tumors. Magnetic fluid hyperthermia can generate heat in an alternating magnetic field by means of physical rotation and magnetic vector rotation, leading to apoptosis and irreparable cellular damage. Su et al. investigated CD44, a well‐known oral carcinoma biomarker that encourages cancer cell immune escape, using anti‐CD44 antibody‐conjugated superparamagnetic iron oxide NPs.^152^ Legge et al.'s invention of biocompatible silica‐coated magnetic iron oxide NPs connected with antibodies has as its goal the overexpression of integrin v6, a biomarker associated with a poor prognosis in oral squamous cell cancer.^153^ MRI is a noninvasive imaging technique that is frequently applied in modern clinical care. As MRI contrast agents that can help with proton relaxation, MNPs are being extensively explored for their potential as efficient probes for both medical and biological diagnostics.^154^ While avoiding other organs, MNPs can potentially be administered directly into the tumor.^155^ Utilizing the particular genetic markers of these conditions, the next generation of active targeting MNPs should significantly improve tumor detection and localization.^156^

### 3.8 Limitations in using NPs in cancer treatment

While nanotechnology has shown great promise in cancer treatment, it also has some limitations that need to be addressed. Here are a few limitations associated with nanotechnology in cancer treatment. NPs used in cancer treatment may face challenges in effectively penetrating solid tumors. The complex tumor microenvironment, including dense extracellular matrix and high interstitial fluid pressure, can hinder the efficient delivery of NPs to the tumor cells. The immune system recognizes NPs as foreign bodies and can clear them from the bloodstream before they reach the tumor site. This clearance can reduce the effectiveness of NP‐based therapies. While NPs can be functionalized with targeting ligands to improve specificity, achieving precise targeting remains a challenge. NPs may encounter off‐target effects, leading to potential toxicity or limited therapeutic efficacy. The long‐term safety of nanomaterials used in cancer treatment is still being studied. Some NPs may have toxic effects on healthy cells or accumulate in organs, causing unintended side effects. The production of NPs at large scales with consistent quality can be challenging. Ensuring reproducibility, scalability, and cost‐effectiveness are important considerations for clinical translation. Nanotechnology‐based therapies face regulatory challenges due to the unique properties of NPs and the need for rigorous safety and efficacy evaluations. Stringent regulatory processes may delay the translation of nanotechnology into clinical practice. Addressing these limitations requires further research and development. Scientists are exploring various strategies such as optimizing NP properties, improving tumor targeting, enhancing NP stability, and evaluating long‐term safety to overcome these challenges and unlock the full potential of nanotechnology in cancer treatment.

## 4 CONCLUSION

The primary approach of treatment for those with oral cancer is chemotherapy, but due to the numerous drawbacks of this medical approach, nanoplatforms are being created. The capability of nanotechnology‐based systems to offer a logical strategy for anticancer therapy has been discussed from this perspective. These nanoplatforms have the potential to overcome many of the drawbacks of chemotherapy, enable drugs to precisely target tumor cells, reduce unfavorable side effects on nearby healthy tissues, and pave the way for the creation of cutting‐edge treatments for oral cancer. Toxicities, instability, and insufficient blood circulation time must all be overcome before using nanoplatforms for the clinical treatment of oral cancer. From nanotechnology, new difficulties for the future of DDSs include the practicality of scaling‐up methods to quickly bring breakthrough therapeutics to society, as well as the possibility of acquiring multifunctional systems capable of meeting various biological and therapeutic criteria.

## AUTHOR CONTRIBUTIONS

**Kalpani Senevirathna**: Conceptualization; resources; visualization; writing—original draft; writing—review and editing. **Shalindu M. Jayawickrama**: Resources; writing—original draft; writing—review and editing. **Yovanthi A. Jayasinghe**: Resources; visualization; writing—original draft; writing—review and editing. **Karunakalage I. P. Prabani**: Resources; writing—original draft; writing—review and editing. **Kushani Akshala**: Resources; writing—review and editing. **Ratupaskatiye G. G. R. Pradeep**: Resources; writing—review and editing. **Hewaratne D. W. T. Damayanthi**: Resources; writing—review and editing. **Kalani Hettiarachchi**: Resources; supervision; writing—review and editing. **Thinley Dorji**: Resources; supervision; writing—review and editing. **Don E. Lucero‐Prisno**: Resources; supervision; writing—review and editing. **Rajapakse M. G. Rajapakse**: Resources; supervision; writing—review and editing. **Kehinde K. Kanmodi**: Resources; writing—review and editing. **Ruwan D. Jayasinghe**: Resources; supervision; writing—review and editing.

## CONFLICTS OF INTEREST STATEMENT

Kehinde K. Kanmodi is an Editorial Board member of Health Science Reports and a coauthor of this article. To minimize bias, they were excluded from all editorial decision‐making related to the acceptance of this article for publication. The remaining authors declare no conflict of interest.

## TRANSPARENCY STATEMENT

The corresponding author Kehinde K. Kanmodi affirms that this manuscript is an honest, accurate, and transparent account of the study being reported; that no important aspects of the study have been omitted; and that any discrepancies from the study as planned (and, if relevant, registered) have been explained.

## Data Availability

Data sharing is not applicable to this article as no new data were created or analyzed in this study.
